# Factors Associated With Prolonged Length of Stay in Adult Clinical Admissions: A Retrospective Cohort Study

**DOI:** 10.1111/jep.70745

**Published:** 2026-11-12

**Authors:** Juliana Ranalli Rinaldi, Silvia Helena Zem Mascarenhas, Daniela Brassolatti, Fabio Fernandes Neves

**Affiliations:** ^1^ Graduate Program in Nursing, Federal University of São Carlos São Carlos São Paulo Brazil; ^2^ University Hospital of the Federal University of São Carlos São Carlos São Paulo Brazil; ^3^ Department of Medicine Federal University of São Carlos São Carlos São Paulo Brazil

**Keywords:** discharge planning, health services evaluation, hospital discharge, length of stay, patient classification

## Abstract

**Background:**

Hospital admissions account for a substantial proportion of healthcare expenditure, motivating the study of factors associated with extended hospitalization and resource use. Routine clinical and care complexity information may be relevant to discharge planning, but whether incorporating these measures into clinical workflows improves patient or service outcomes requires prospective evaluation.

**Objective:**

To examine the associations of early clinical and functional measures and events occurring during hospitalization with prolonged length of stay among adult clinical admissions to a public university hospital, including the contributions of two structured assessment instruments.

**Methods:**

This retrospective cohort study analysed 250 clinical admissions at a Brazilian university hospital. Prolonged length of stay was defined according to Brazilian Unified Health System criteria. Associations between the Blaylock Risk Assessment Screening Score, Fugulin Scale, and prolonged length of stay were examined using simple and multivariable logistic regression. Apparent model discrimination was assessed using receiver operating characteristic analysis; no internal validation was performed.

**Results:**

Prolonged length of stay occurred in 39.6% of admissions. Among these, excess days beyond expected length of stay represented 30% of all bed days. The Fugulin Scale remained associated with prolonged length of stay in the adjusted model (odds ratio = 1.14; 95% confidence interval, 1.05–1.25), whereas the Blaylock Risk Assessment Screening Score was no longer significant after adjustment (*p* = 0.390). Intensive care unit admission and genitourinary diagnoses were also associated with prolonged length of stay (odds ratios of 2.85 and 6.08, respectively). Healthcare‐associated infections were recorded in only 11 admissions; although their adjusted odds ratio was 7.10, the wide 95% confidence interval (1.22–41.22) indicates substantial uncertainty. The model had an area under the curve of 0.783 and a Nagelkerke *R* ^2^ of 0.307. Because intensive care unit admission and healthcare‐associated infections may occur after the initial assessment, these performance estimates do not represent admission‐time prediction.

**Conclusion:**

The Fugulin Scale score was associated with prolonged length of stay in this retrospective analysis, while intensive care unit admission and healthcare‐associated infections reflected events in the evolving hospital course. Whether using the Fugulin Scale improves prediction, discharge planning, or resource allocation requires prospective evaluation. The combined model should not be used as an admission‐time prediction tool.

## 1 Introduction

Hospital care accounts for a substantial share of healthcare expenditure, and rising costs have increased pressure on health systems to improve efficiency without compromising quality. Within this context, the optimization of inpatient processes has become a central concern in health services research [1]. Length of stay (LoS) is a widely used indicator of hospital efficiency, patient flow and resource use. However, its interpretation requires caution because it reflects the interaction between case mix, patient complexity, clinical processes and organizational constraints. When not clinically justified, prolonged LoS (PLoS) is associated with higher costs, reduced bed turnover and restricted access to acute care [2].

These challenges are particularly relevant in Brazil within the Sistema Único de Saúde (SUS), a universal public health system operating under persistent capacity and resource constraints. Efforts to improve efficiency and control costs coexist with increasing pressure on hospital capacity. A 2022 study reported the loss of 35,080 SUS hospital beds between 2011 and 2019, indicating a sustained reduction in inpatient availability [3]. In this setting, extended hospitalization has consequences beyond individual care, affecting service organization and the timely availability of beds for other patients requiring admission.

LoS is a multifactorial outcome shaped by determinants at several levels, and the relevance of individual factors may vary across clinical populations, underlying diseases and patient subgroups. Patient‐related factors include disease severity, primary diagnosis, comorbidity burden, cognitive impairment, socioeconomic disadvantage, nutritional status and functional dependence. For example, a study of older adults hospitalised with heart failure examined how different definitions of obesity relate to LoS, while a study of patients with atrial fibrillation found that nutritional risk was associated with longer hospitalization in men but not in women [4, 5, 6]. Care‐related factors include ward type, clinician experience, patient safety practices and complications occurring during admission, whereas external determinants include cultural barriers to discharge and limited access to post‐acute care. This heterogeneity supports examining diagnostic profile alongside functional status and care‐related characteristics when assessing the risk of prolonged hospitalization [7].

In this context, information available early in hospitalization may help identify patients who need case management and interdisciplinary discharge planning [8]. As clinical status and care needs can change, subsequent events may also inform reassessment throughout the hospital stay. From an evaluation perspective, it is essential to distinguish the potential value of structured clinical and functional instruments used early in admission from the information provided by events that occur later.

Structured assessment tools systematically describe dimensions relevant to hospitalization and discharge, including comorbidity, functional dependence and care complexity. Their association with LoS may help identify factors for further investigation, although clinical utility requires evaluation beyond an analysis of routinely collected records.9 Evidence on the relationship between these measures and PLoS among adult clinical inpatients in Brazil remains limited. In this retrospective study, we aimed to examine associations of demographic characteristics, diagnostic profile, early clinical and functional measures, and events occurring during hospitalization with PLoS in a public university hospital. We also examined the adjusted associations of the Blaylock Risk Assessment Screening Score (BRASS) and the Fugulin Scale, distinguishing measures available early from markers of the subsequent hospital course. Developing or validating a tool to predict PLoS prospectively at admission was not an objective of this study.

## 2 Methods

### 2.1 Study Design and Setting

This retrospective cohort study used routinely collected electronic health record data from adult clinical admissions between December 1, 2024, and February 28, 2025. The report was structured in accordance with STROBE recommendations for observational studies. The study was approved by the Research Ethics Committee of the Federal University of São Carlos (CAAE: 59100622.3.0000.5504), with a waiver of informed consent.

The study was conducted at the University Hospital of the Federal University of São Carlos, located in São Carlos, São Paulo state, which provides care exclusively through the SUS. The hospital has 80 active inpatient beds, including 20 allocated to internal medicine. This context is relevant because bed availability and discharge processes may influence LoS independently of patient‐level factors. Admissions occur mainly through the emergency unit, either via the State of São Paulo Computerised Regulation System or directly through the Mobile Emergency Care Service. The SUS Procedures Management Table specifies an expected duration of hospitalization for each procedure [9]. We defined PLoS as a stay exceeding twice the expected duration for the corresponding procedure. This two‐fold threshold was chosen because it corresponds to the threshold used in SUS hospital information rules for recording additional days of stay beyond the expected procedure‐specific duration. It therefore provides a locally relevant measure of substantially extended hospitalization for service evaluation, rather than a universally established clinical definition of PLoS.

### 2.2 Participants

All admissions to the internal medicine unit during the study period were eligible. The unit of analysis was the hospital admission. Repeated admissions by the same patient were retained as separate admission events, and patient identifiers were used to determine the number of unique patients and repeated admissions. The regression analysis did not account for clustering of admissions within patients.

Total LoS was calculated from hospital entry to discharge and included time spent in the emergency unit before ward transfer, and any period in the intensive care unit (ICU). Specialised admissions, including those for mental health treatment and tuberculosis care, were excluded. One admission involving judicial discharge and a 509‐day hospital stay was excluded because it represented an extreme administrative outlier driven by legal and social factors rather than by usual clinical discharge processes.

### 2.3 Assessment Instruments

Two instruments were used to assess clinical, functional and care‐related factors potentially associated with PLoS: the BRASS and the Fugulin Scale.

The BRASS was applied using a free translation because no validated cross‐cultural adaptation for Brazilian Portuguese was available. This limitation was considered when interpreting the instrument's performance, as measurement equivalence cannot be assumed. The instrument was originally developed to identify patients at risk of prolonged hospitalization and complex discharge planning [10]. It includes 10 domains: age, social support, functional status, cognition, behavioural pattern, mobility, sensory deficits, previous admissions, number of active diagnoses and medications in use. Scores range from 0 to 40, based on which patients are classified as being at low, moderate or high risk.

The Fugulin Scale was used to assess patient care complexity and nursing workload [11]. As it is routinely recorded by nursing staff in the study hospital, it represents a pragmatic measure of care dependency routinely available in this setting.

It evaluates 12 care areas: mental status, oxygenation, vital signs, mobility, ambulation, nutrition, personal care, elimination, therapy, skin integrity and tissue impairment, dressings and dressing time. Scores classify patients as requiring minimal, intermediate, high‐dependency, semi‐intensive or intensive care [12].

### 2.4 Data Collection

Patient data were collected using a standardised form within 48 h of admission to the internal medicine unit. Variables included age, sex, ethnicity, marital status, city of origin, educational level, International Classification of Diseases (ICD‐10) code, and admission date and procedure or treatment code.

Data were extracted from the electronic health record system, including nursing, medical and multidisciplinary team notes. Information required to calculate the BRASS was obtained from records describing activities of daily living, functional status, cognition, behavioural patterns and mobility. These records reflected the patient's clinical condition during hospitalization and were not assumed to represent baseline function in the home.

The Fugulin Scale score was obtained from the nursing assessment logged in the electronic health record within 48 h of ward admission. This tool is routinely used by nursing staff to organise care processes and allocate resources according to patient dependency.

Readmissions were assessed over the preceding 90 days and counted as admission events rather than as unique readmitted patients. Intensive care unit (ICU) admission was identified from bed allocation records and could occur after the initial ward assessment. Palliative care status was determined from medical prescription records. Discharge type and LoS were obtained from discharge records. Thus, the variables analysed were not all necessarily available at the same point in the hospital stay.

Healthcare‐associated infections (HAIs) were identified in consultation with the infection prevention and control team. Cases were detected by active surveillance according to institutional criteria. Infections occurring after 48 h of hospitalization were classified as HAIs, whether they were related to invasive devices. Most recorded infections were bloodstream infections, although Clostridioides difficile infections were also observed.

Data were entered into Microsoft Excel for organization and quality control before import into the statistical software. Data consistency cheques and missing data assessments were performed before analysis. When variables were unavailable in the electronic health record, they were treated as missing and excluded from analyses involving that variable.

### 2.5 Outcomes and Variables

The primary outcome was PLoS, operationally defined as a stay exceeding twice the expected LoS specified for the corresponding procedure in the SUS Procedures Management Table. Admissions at or below this threshold were classified as having a non‐prolonged stay for the purposes of this analysis. This classification reflects an administrative benchmark and does not establish whether an individual hospital day was clinically necessary. LoS was also analysed as a continuous variable.

Categorical variables included sex, city of origin, ethnicity, educational level, marital status, diagnosis group, discharge type, readmission, HAIs, palliative care and ICU admission. Diagnoses were grouped according to the most prevalent ICD‐10 chapters. Discharge type was categorised as medical discharge, death, or transfer to another hospital.

Continuous variables included age and LoS. Ordinal variables included the BRASS, the Fugulin Scale score and the Charlson Comorbidity Index (CCI).

### 2.6 Statistical Analysis

The sample size was determined by the number of eligible admissions during the study period, and no formal a priori sample size calculation was performed. The study included 250 admissions, of which 99 met the definition of PLoS. Because the multivariable model included several predictors, the number of outcome events may have been insufficient for stable estimation of all coefficients. The analysis was therefore considered exploratory and hypothesis‐generating.

Statistical analyses were performed using Jamovi, version 2.6.44, a graphical user interface built on the R statistical engine. Categorical variables were described as absolute and relative frequencies. Continuous and ordinal variables were summarised as medians and interquartile ranges.

Patients with appropriate and prolonged stays were compared using the chi‐square test for categorical variables and the Mann–Whitney *U* test for continuous variables. Statistical significance was set at *p* < 0.05, with 95% confidence intervals (CIs). Boxplots were used to visualise the distribution of instrument scores according to LoS categories.

Associations between assessment scores and PLoS were examined using logistic regression. Variables with *p* < 0.20 in the bivariate analysis were considered for inclusion in the multivariable model as an exploratory screening criterion. Age, sex and race were also included regardless of their bivariate p‐values because they were considered clinically relevant demographic characteristics that could influence the associations under study. Model parsimony was prioritised because of the number of PLoS events. A multivariable logistic regression model was then fitted, and its apparent discrimination and explanatory capacity in the study sample were summarised using the receiver operating characteristic curve and Nagelkerke *R* ^2^. No internal validation, such as bootstrap resampling or cross‐validation, was performed to estimate optimism in model performance. Potential multicollinearity among the BRASS total score, Fugulin Scale score and Charlson Comorbidity Index (CCI) was assessed using variance inflation factors (VIFs) and their corresponding tolerance values in the logistic regression model. The VIFs were 3.81, 3.44 and 1.44, respectively, with tolerance values of 0.262, 0.290 and 0.693. Because the model included ICU admission and healthcare‐associated infections, which could occur after the initial assessment, it was interpreted as an analysis of associations across the hospital course rather than as an admission‐time prediction model.

## 3 Results

### 3.1 Sample Characteristics

The study included 250 admissions involving 229 unique patients. Eighteen patients contributed more than 1 admission: 15 had 2 admissions and 3 had three, yielding 21 admissions beyond their first admission. The median age across admissions was 72.5 years, and 133 admissions involved male patients (53.2%). Most admissions involved patients who self‐identified as White (68.8%), had completed up to primary education (75.2%), and lived in São Carlos (82.8%). Overall, 26.0% of admissions involved patients receiving palliative care, 15.6% involved patients who had been readmitted during the previous 90 days, 4.4% were associated with HAIs, and 21.6% required ICU admission.

The most frequent primary diagnoses were respiratory diseases (ICD‐10 chapter J; 27.7%), infectious and parasitic diseases (chapter A; 16.1%), circulatory diseases (chapter I; 14.1%) and genitourinary diseases (chapter N; 13.7%). The median LoS was 6.5 days. Regarding clinical and functional indices, the median Fugulin Scale score was 20.0, the median BRASS score was 12.5, and the median CCI was 4.5, indicating a population characterised by older age, substantial care needs and a moderate comorbidity burden.

### 3.2 Comparison Between Adequate and Prolonged Stay Groups

Of the 250 admissions, 151 (60.4%) were classified as having a non‐prolonged stay and 99 (39.6%) as having PLoS. Demographic characteristics were comparable between groups, with no statistically significant differences in age, sex, race, marital status, educational level or city of residence. By contrast, hospital‐related complications, care intensity, and clinical and functional scores differed between groups. Admissions with PLoS had higher frequencies of HAIs and ICU care, and higher BRASS and Fugulin Scale scores, suggesting greater care complexity and a more complicated hospital course. Palliative care, mortality and CCI values were numerically higher in the PLoS group but did not reach statistical significance, whereas readmission within the previous 90 days did not differ between groups.

Primary diagnosis also varied according to LoS classification. Genitourinary diseases were more frequent among admissions with PLoS, and overall differences were observed across the main ICD‐10 diagnostic groups. As diagnostic groups were analysed as categories of the same variable, the p‐value refers to the overall comparison across groups. These patterns are summarised in Table 1 and illustrated in Figure 1, which shows higher BRASS and Fugulin Scale score distributions among admissions with PLoS.

**Table 1:** Comparison of patient characteristics between the appropriate and prolonged length‐of‐stay groups.

	Participants		
Variable	Adequate stay (*n* = 151)	PLoS (*n* = 99)	*p*‐value
**Demographic data**			
Age [median (IQR)]	71 (24.5)	75 (22.0)	0.255
Male sex [*n* (%)]	82 (54.3)	51 (51.5)	0.666
White race [*n* (%)]	105 (69.5)	67 (67.7)	0.756
Married/cohabiting (%)	74 (49.7)	45 (46.4)	0.616
Up to primary education [*n* (%)]	110 (72.8)	78 (78.8)	0.287
Residing in São Carlos [*n* (%)]	125 (82.8)	82 (82.8)	0.992
**Hospital conditions**			
Readmission [*n* (%)]	24 (15.9)	15 (15.2)	0.874
HAIs [*n* (%)]	2 (1.3)	9 (9.1)	**0.003**
Palliative patient [*n* (%)]	33 (21.9)	32 (32.3)	0.065
ICU admission [*n* (%)]	24 (15.9)	30 (30.3)	**0.007**
**Clinical and functional indices**			
BRASS [median (IQR)]	10 (16.0)	18 (15.0)	**0.013**
Fugulin [median (IQR)]	18 (10.5)	23 (11.5)	**< 0.001**
CCI [median (IQR)]	4 (3.5)	5 (2.5)	0.076
**Primary diagnosis (ICD)**			
J (Respiratory system) [*n* (%)]	42 (27.8)	27 (27.3)	**< 0.001**
A (Infectious and parasitic) [*n* (%)]	30 (19.8)	10 (10.1)	
I (Circulatory system) [*n* (%)]	23 (15.2)	12 (12.1)	
N (Genitourinary system) [*n* (%)]	8 (5.3)	26 (26.2)	
**Outcomes**			
Death [*n* (%)]	13 (8.6)	16 (16.2)	0.068
LOS, days [median (IQR)]	6.0 (5.0)	7.50 (6.0)	0.560

Abbreviations: BRASS, Blaylock Risk Assessment Screening Score; CCI, Charlson Comorbidity Index; HAIs, healthcare‐associated infections; ICD, International Classification of Diseases; IQR, interquartile range; LoS, length of stay; PLoS, prolonged length of stay.

**Figure 1:**
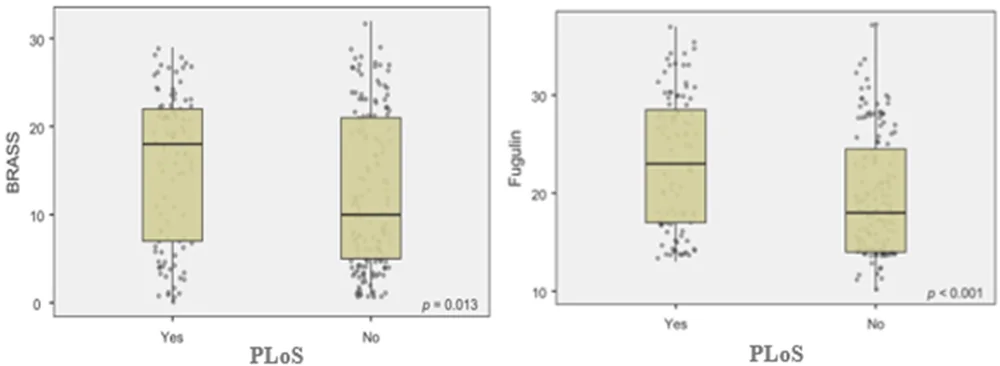
Distribution of BRASS and Fugulin Scale scores according to PLoS status. Boxplots showing the distribution of Blaylock Risk Assessment Screening Score (BRASS) and Fugulin Scale scores among admissions with and without prolonged length of stay (PLoS).

### 3.3 Association Between Assessment Scores and PLoS

In bivariate logistic regression, higher BRASS and Fugulin Scale scores were associated with an increased probability of PLoS, with respective *p*‐values of 0.008 and < 0.001. These findings indicate that greater discharge complexity and higher care dependency were associated with a higher likelihood of extended hospitalization. The regression plots showed a progressive increase in the fitted probability of PLoS as BRASS and Fugulin Scale scores increased (Figure 2).

**Figure 2:**
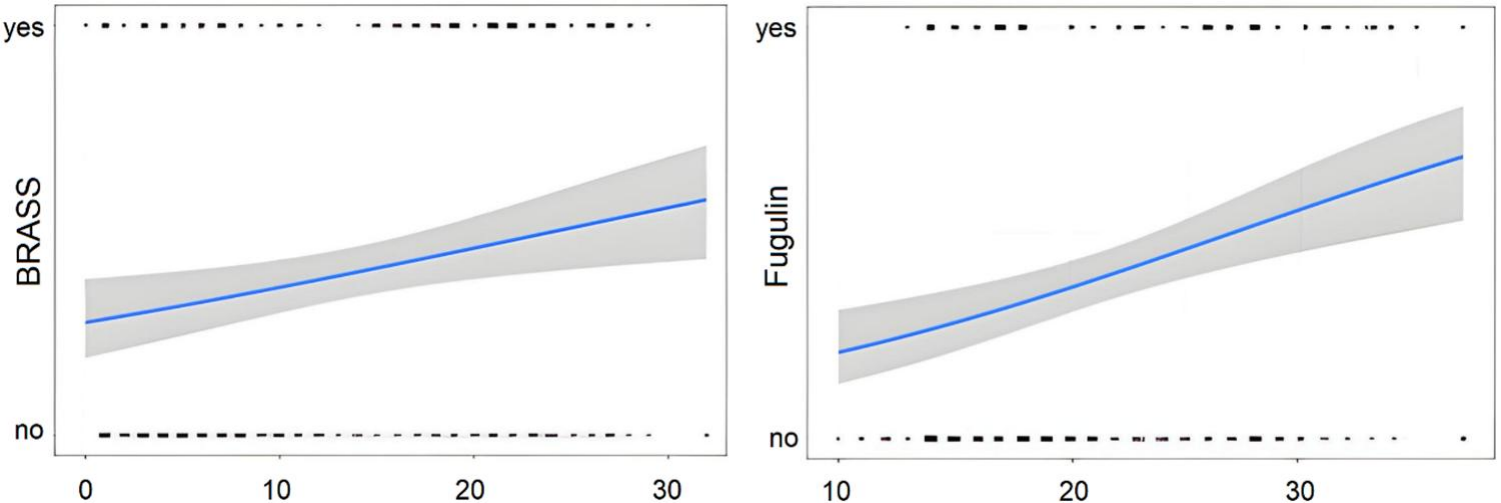
Bivariate logistic regression of BRASS and Fugulin Scale scores for prolonged length of stay. BRASS = Blaylock Risk Assessment Screening Score. Dots represent individual admissions classified as having or not having prolonged length of stay. The fitted lines indicate the estimated probability of prolonged length of stay, with shaded areas representing 95% confidence intervals.

In the adjusted model, ICU admission, ICD‐10 diagnostic group and the Fugulin Scale score were associated with PLoS. ICU admission was associated with nearly threefold higher odds of PLoS (odds ratio [OR] = 2.85; 95% CI: 1.32–6.14; *p* = 0.007). HAIs occurred in 11 admissions, including 9 classified as PLoS. The adjusted association between HAIs and PLoS was large (OR = 7.10; 95% CI: 1.22–41.22; *p* = 0.029), but the small number of infections and wide CI indicate substantial statistical uncertainty; the magnitude of this association should not be considered a precise or robust estimate. Compared with respiratory diseases, genitourinary conditions were associated with higher odds of PLoS (OR = 6.08; 95% CI: 2.22–16.68; *p* < 0.001). Each 1‐point increase in the Fugulin Scale score was associated with 14% higher odds of PLoS (OR = 1.14; 95% CI: 1.05–1.25; *p* = 0.002). ICU admission and HAIs should be interpreted as markers of the hospital course, as they may not have been present at the time of the initial assessment.

In contrast, palliative care, BRASS and CCI were not independently associated with PLoS after adjustment. The Nagelkerke *R* ^2^ was 0.307, indicating a moderate explanatory capacity.

### 3.4 Discriminatory Performance of the Model

The multivariable logistic regression model showed an apparent area under the receiver operating characteristic curve of 0.783 (Figure 3), with an accuracy of 73.5%, specificity of 86.8% and sensitivity of 53.1%. These estimates were obtained from the data used to fit the model and were not corrected for optimism through internal validation. Because the model also includes events occurring during hospitalization, they should not be interpreted as measures of predictive performance at admission.

**Figure 3:**
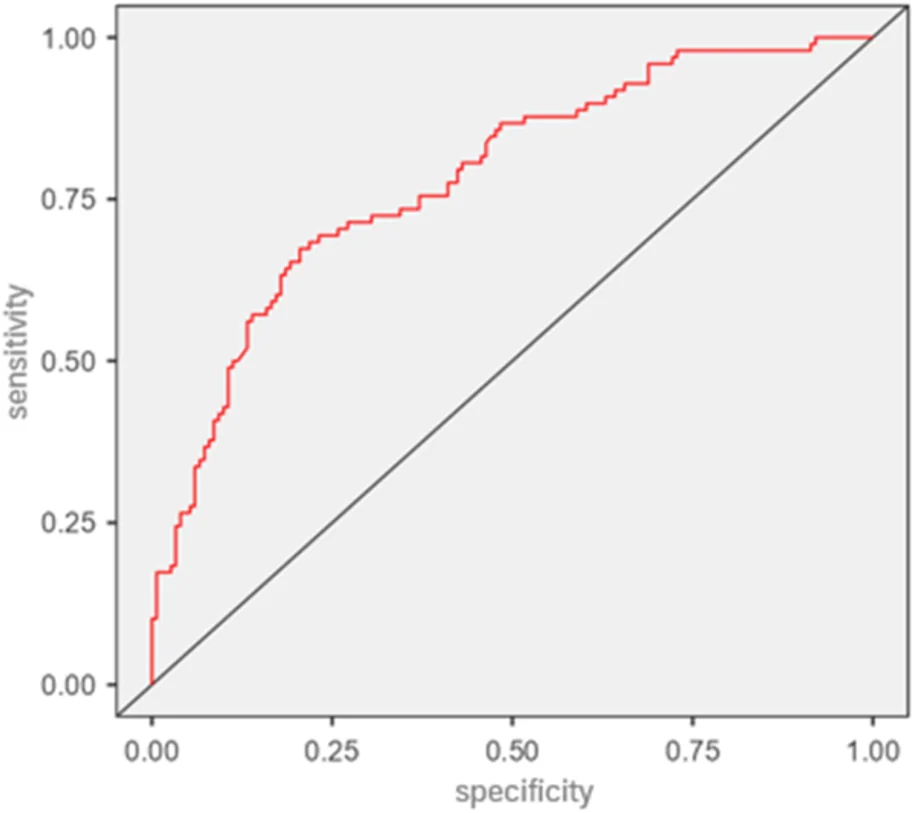
Receiver operating characteristic curve of the multivariable model for prolonged length of stay.

These findings suggest that the model was more effective in identifying admissions unlikely to develop PLoS than in detecting all admissions that would exceed the expected LoS, a pattern reflected by high specificity and moderate sensitivity. This pattern is consistent with the multifactorial nature of hospitalization duration, which depends not only on baseline clinical and functional characteristics but also on events occurring during admission, such as ICU care and HAIs.

## 4 Discussion

This retrospective study examined factors associated with PLoS among adult clinical admissions to a public university hospital in Brazil, including measures available early in hospitalization and events recorded during the subsequent hospital course. Nearly two in five admissions exceeded the expected duration of hospitalization according to SUS criteria. In the adjusted analysis, ICU admission, genitourinary diagnoses, and the Fugulin Scale were associated with PLoS, whereas the BRASS did not remain statistically significant after adjustment. HAIs were also associated with PLoS, but this finding was based on only 11 infections and had a wide confidence interval, warranting cautious interpretation. Because the analysis included events occurring after admission and the model was not internally validated, its results primarily describe associations within this sample. They do not establish how accurately PLoS could be predicted at admission or support use of the fitted model as a clinical prediction tool.

The bivariate association between the BRASS and PLoS is consistent with previous studies showing that a higher BRASS is related to longer hospitalization and greater discharge complexity. The BRASS did not retain an independent association in the adjusted model, which also included measures of care dependency and clinical characteristics. This finding must be interpreted considering the instrument's free translation: without a validated Brazilian Portuguese adaptation, it is unknown whether the translated items measure the intended domains equivalently to the original scale. Measurement error may therefore have weakened the observed association. The VIFs for BRASS and the Fugulin Scale were 3.81 and 3.44, respectively, indicating some shared information between these measures but not severe multicollinearity. Thus, both measurement limitations and overlap between instrument domains may have contributed to the adjusted result; it should not be taken as evidence that the BRASS has less intrinsic clinical value than the Fugulin Scale. This interpretation is compatible with reports by Hodgins et al., Dal Molin et al. and Mistiaen et al., who found associations between BRASS risk categories and LoS or discharge‐related outcomes while also noting limitations in sensitivity or predictive performance for some clinically relevant endpoints [13, 14, 15].

The Fugulin Scale remained associated with PLoS after adjustment. Although originally designed to support nursing workload assessment and patient classification, its score may reflect clinical vulnerability, functional dependency, and intensity of care in this hospital setting. Freitas et al. also found an association between care complexity assessed using the Fugulin classification system and LoS among hospitalised tube‐fed patients [16]. Patients requiring greater nursing input might need more complex discharge arrangements, including caregiver support, family training, equipment, home adaptations and coordination with community services; however, this proposed explanation was not tested in our study. Our retrospective analysis examined associations and was not designed to determine whether implementing the Fugulin Scale improves prediction, discharge planning or resource allocation. Moreover, the different adjusted associations of Fugulin and BRASS do not establish that one instrument is intrinsically superior, particularly because the BRASS was used without a validated Brazilian Portuguese adaptation.

These results also align with the broader evolution of BRASS‐based approaches. Zarovska et al. developed and validated a simplified BRASS index to screen hospitalised patients requiring personalised discharge planning, showing that selected components could predict complex discharge and in‐hospital mortality, although discrimination for complex discharge was only moderate [10]. Together with our findings, this suggests that the BRASS may be useful as an early screening instrument for discharge complexity, but assessment of PLoS risk probably requires integration with variables reflecting care dependency, complications and diagnostic categories.

ICU admission and HAIs may reflect changes in clinical status during hospitalization and should be interpreted as markers of the hospital course rather than as baseline predictors. Previous studies have associated in‐hospital adverse events with longer LoS and higher mortality and have linked HAIs to excess hospital stay in different settings [17, 18, 19]. In the present study, however, only 11 admissions involved HAIs. Although the adjusted OR was elevated, its wide confidence interval is compatible with a broad range of effect sizes, and the estimate may be sensitive to a small number of observations. This finding is therefore exploratory and should not be taken as evidence of a stable independent effect or of causation. It nevertheless reinforces the clinical importance of infection prevention and monitoring for complications during hospitalization.

Genitourinary diagnoses were also independently associated with PLoS. This finding may reflect the complexity of urinary and renal conditions requiring inpatient care, including complicated urinary tract infections, obstructive uropathy, renal dysfunction and infection‐related complications. Studies of hospitalised patients with urinary tract infections have reported longer LoS in the presence of comorbidity, functional dependence, bacteremia, resistant pathogens and complicated infection patterns [20, 21]. In this sense, the diagnostic category may act as a proxy for a cluster of clinical conditions that frequently require prolonged stabilization, antimicrobial therapy, device management or coordination of post‐acute care. This interpretation should be considered exploratory because diagnoses were grouped by ICD‐10 chapter rather than by specific clinical syndromes.

The timing of each variable is central to interpreting these findings. Demographic characteristics, diagnostic profile, and early measures of care complexity were available near the beginning of hospitalization, whereas ICU admission and HAIs could arise later. The combined model had higher apparent specificity than sensitivity in the study sample, but these measures were calculated without internal validation and include information from the subsequent hospital course. They therefore describe the fitted model in these data rather than its prospective predictive performance. Evaluating prediction at admission would require a separately developed model restricted to information available at a defined early time point, followed by internal and external validation.

Several considerations inform the interpretation of these findings. The retrospective, single‐centre design relied on routinely documented clinical data, and the sample comprised all eligible admissions during the study period, without a formal sample size calculation before data collection. With 99 PLoS events and several model parameters, some adjusted estimates may be unstable, particularly the HAI estimate, which was based on few infections and had a wide confidence interval. The BRASS was applied using a free translation without established measurement equivalence in Brazilian Portuguese; its lack of an adjusted association therefore does not establish lower clinical value than the Fugulin Scale. VIF assessment did not indicate severe multicollinearity among the three scores examined, although BRASS and Fugulin shared some information. PLoS was defined using a locally relevant SUS administrative threshold, which may limit comparison with other settings. Finally, ICU admission and HAIs could arise after the initial assessment, and the model was neither restricted to early information nor internally validated. Its reported discrimination should therefore be viewed as apparent performance within this sample rather than evidence of admission‐time predictive utility. Within these bounds, the study identifies associations that can guide prospective evaluation of care complexity and hospitalization duration.

Despite these limitations, this study contributes to the evaluation of clinical practice by examining associations between two structured instruments used in routine hospital care and PLoS. The adjusted association observed for the Fugulin Scale suggests that nursing care complexity warrants further study as a component of assessment during hospitalization. Whether incorporating this measure into early discharge planning improves decisions, bed management, or resource allocation was not evaluated here. These potential clinical implications should be tested prospectively.

### 4.1 Conclusion

In this retrospective study, PLoS was associated with the Fugulin Scale score, diagnostic profile and ICU admission in the adjusted analysis. HAIs were also associated with PLoS, although the estimate was imprecise because infections were uncommon. The BRASS did not retain an independent association, but its use through a free translation without established measurement equivalence precludes concluding that it has lower clinical value than the Fugulin Scale.

The analysis was designed to examine associations, not the effects of implementing a screening instrument. Because the model included events arising during hospitalization and was not internally validated, it should not be used to predict PLoS at admission. Prospective studies should assess measures available at clearly defined time points and test whether incorporating care complexity assessment into clinical workflows improves discharge planning, bed management or resource allocation.

## Author Contributions

**Juliana Ranalli Rinaldi:** conceptualization, data curation, formal analysis, investigation, methodology, writing and original draft. **Silvia Helena Zem Mascarenhas:** supervision, review and editing. **Daniela Brassolatti:** data curation, investigation. **Fabio Fernandes Neves:** conceptualization, methodology, supervision, writing, review and editing.

## Funding

The authors have nothing to report.

## Conflicts of Interest

The authors declare no conflicts of interest.

## Data Availability

The data that support the findings of this study are available on request from the corresponding author.
